# Crosstalk between long non‐coding RNAs and Wnt/β‐catenin signalling in cancer

**DOI:** 10.1111/jcmm.13522

**Published:** 2018-02-01

**Authors:** Gang Yang, Tianyi Shen, Xiaoming Yi, Zhengyu Zhang, Chaopeng Tang, Longxin Wang, Yulin Zhou, Wenquan Zhou

**Affiliations:** ^1^ Department of Urology Jinling Hospital Nanjing Medical University Nanjing Jiangsu Province China; ^2^ Department of Urology Jinling Hospital School of Medicine Nanjing University Nanjing Jiangsu Province China

**Keywords:** cancer, long non‐coding RNA, Wnt/β‐catenin signalling pathway

## Abstract

Long non‐coding RNAs (lncRNAs) are non‐protein‐coding transcripts in the human genome which perform crucial functions in diverse biological processes. The abnormal expression of some lncRNAs has been found in tumorigenesis, development and therapy resistance of cancers. They may act as oncogenes or tumour suppressors and can be used as diagnostic or prognostic markers, prompting their therapeutic potentials in cancer treatments. Studies have indicated that many lncRNAs are involved in the regulation of several signal pathways, including Wnt/β‐catenin signalling pathway, which has been reported to play a significant role in regulating embryogenesis, cell proliferation and controlling tumour biology. Emerging evidences have suggested that lncRNAs can interact with several components of the Wnt/β‐catenin signalling pathway to regulate the expression of Wnt target genes in cancer. Moreover, the expression of lncRNAs can also be influenced by the pathway. Nevertheless, Wnt/β‐catenin signalling pathway‐related lncRNAs and their interactions in cancer are not systematically analysed before. Considering these, this review emphasized the associations between lncRNAs and Wnt/β‐catenin signalling pathway in cancer initiation, progression and their therapeutic influence. We also provided an overview on characteristics of lncRNAs and Wnt/β‐catenin signalling pathway and discussed their functions in tumour biology. Finally, targeting lncRNAs or/and molecules associated with the Wnt/β‐catenin signalling pathway may be a feasible therapeutic method in the future.

## 1 INTRODUCTION

Cancer is a class of disease with the potential to proliferate continuously due to stepwise alterations at the genetic, epigenetic and cellular levels. It has become a great threat to human health. Though having been extensively studied, multidrug resistance (MDR) of tumour cells remains to be an obvious barrier of cancer treatments. Therefore, a better understanding of mechanisms in tumorigenesis, progression and drug resistance in cancer will be imperative for the development of efficient therapeutic strategies. Recently, more and more reports revealed that mutations within the non‐coding genome have a close relationship with tumour biology.^1^

Results from the latest GENCODE have indicated that there are only approximately 2% of the human genome are protein‐coding genes, whereas the vast majority of the human genome is made up of non‐coding RNAs (ncRNAs).^2^ In the past decades, lots of ncRNAs have been found, such as ribosomal RNA (rRNA), transfer RNA (tRNA), small nuclear RNA (snRNA) and microRNA (miRNA) as well as long non‐coding RNA (lncRNA). Aberrations within the non‐coding genome are major decisive factors contributed to human diseases.^3^ Among these non‐protein‐coding transcripts, lncRNAs, defined as longer than 200 nucleotides with little or no protein‐coding ability, have emerged as essential regulators in diverse biological processes.^4^, ^5^, ^6^ They can interplay with DNA, RNA and proteins to regulate the target gene expression. They can also influence several signalling pathways including Wnt/β‐catenin signalling pathway, which is a common pathway involved in embryogenesis, development and homoeostasis of tissues. Aberrant regulation of the pathway is associated with human diseases.^7^, ^8^

The comprehensive interrelationship of lncRNAs and Wnt/β‐catenin signalling pathway has appeared as a significant tumorigenic signalling network in various cancers. In this review, we summarized some lncRNAs which are associated with Wnt/β‐catenin signalling pathway to describe their interactions and underlying mechanisms in cancer formation, development and response to treatments.

## 2 CHARACTERISTICS OF LNCRNAS AND THEIR FUNCTIONS IN CANCER

LncRNAs represent a novel heterogeneous group of ncRNAs that usually exhibit cell‐type–specific expression manner.^9^ The genomic locations of lncRNAs can be classified into intragenic, bidirectional and intergenic on the basis of their relationship to adjacent protein‐coding transcripts.^10^ They are initially transcribed through pathways similar to that of protein‐coding genes. And their transcription is mediated via RNA polymerase II.^11^, ^12^ Evidences have already indicated that lncRNAs can regulate gene expression at the epigenetic, transcriptional and post‐transcriptional levels.^13^ For example, lncRNA CCAT2, which is up‐regulated in colorectal cancer (CRC), can enhance tumour invasion and metastasis by regulating c‐Myc transcription and activate the Wnt signalling pathway.^14^ LncRNA CCAT1 can promote the progression of gastric carcinoma depending on the post‐transcriptional activity of c‐Myc.^15^

Functionally, lncRNAs are not the “transcriptional junk” any more. They play an important role in normal cellular proliferation and differentiation.^16^ They are predominantly localized in the nucleus or cytoplasm, and their function is location‐specific.^17^ In nucleus, lncRNAs are involved in chromatin remodelling, transcriptional regulation and RNA processing, while cytoplasmic lncRNAs can modulate mRNA stability, splicing or translation regulation, interact with special proteins and influence cellular signalling cascades.^18^ Studies have shown that dysregulated expression of lncRNAs leads to diverse human diseases.^19^, ^20^, ^21^ At present, masses of lncRNAs are known to be associated with the initiation and progression of cancer. They can function as oncogenes or tumour suppresses.^22^ Many lncRNAs have a tissue and cancer type–specific expression pattern and have already been shown to be useful as diagnostic and prognostic markers.^23^, ^24^, ^25^ More recently, emerging evidences have revealed an important role of lncRNAs in tumour drug resistance as well.^26^ These findings strongly suggest that they could become potential therapeutic targets in cancer treatments.

## 3 Wnt/β‐CATENIN SIGNALLING PATHWAY AND ITS ROLE IN CANCER

Wnt signalling pathway is an evolutionarily highly conserved pathway which controls critical biological processes including embryonic development, normal homoeostasis of adult tissues and cell fate.^27^, ^28^ It mainly contains three diverse signalling pathways: Wnt/β‐catenin signalling pathway (canonical pathway), Wnt/Ca^2+^ pathway and Wnt/planar cell polarity (PCP) pathway. Among them, Wnt/β‐catenin signalling pathway (Wnt pathway for short) has obtained particular attentions due to its versatile functions in cells. In this section, we will provide an overview of the molecular mechanism of Wnt pathway.

**Figure 1:**
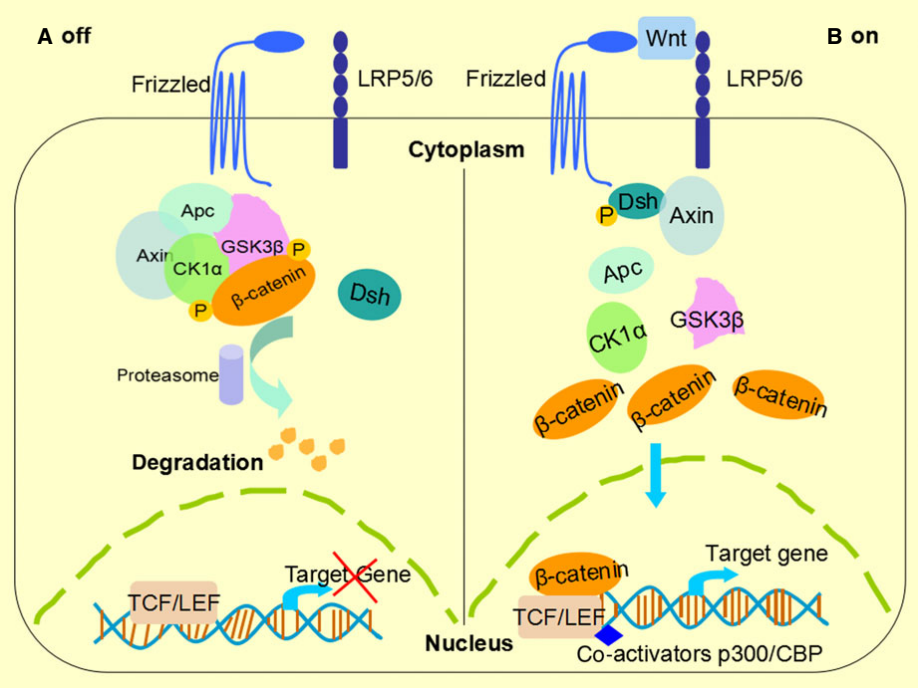
The Wnt/β‐catenin signalling pathway. (A) WNT OFF: Wnt ligands do not bind to Frizzled/Lrp5/6 receptors complex on the surface of cells. β‐catenin is recruited by the degradation complex, including APC, Axin, GSK3β and CK1α, which phosphorylates ß‐catenin and targets it for proteasome‐mediated degradation. (B) WNT ON: In the presence of Wnt ligands, the receptor complex sends signal to Dsh (Dishevelled) and Axin, resulting in the destruction of degradation complex and accumulation of ß‐catenin in the cytoplasm. Then ß‐catenin translocates into nucleus, where it can interact with TCF/LEF and other co‐activators such as CBP and p300 to control the transcription of target genes

The primary components of Wnt pathway are as follows: Wnt ligands, Frizzled receptors, LRP5/6 co‐receptors (low density lipoprotein receptor‐related protein 5/6), Dsh (Dishevelled), β‐catenin and TCF/LEF (T‐cell factor/lymphoid enhancer factor) as well as one “degradation complex” composed of APC (adenomatous polyposis coli), axin (the scaffolding protein) and two kinases: CK1α (casein kinase 1α) and GSK‐3β (glycogen synthase kinase 3β). Wnt ligand and β‐catenin play a vital role in this pathway. In the absence of Wnt ligand, the signalling cascade is repressed. Cytoplasmic β‐catenin is phosphorylated and kept in low levels via proteasome‐mediated destruction, which is controlled by the “degradation complex”. As a result, the translocation into nucleus is inhibited. However, when Wnt ligand binding to receptors at the surface of target cells, a chain of events is initiated that disrupt the degradation complex via Dsh (Dishevelled) phosphorylation. Then β‐catenin is separated from the destruction complex and results in the accumulation and stabilization of β‐catenin in cytoplasm.^29^, ^30^ Subsequently, β‐catenin is imported into the nucleus where it can interact with the TCF/LEF family transcription factors and recruit transcriptional co‐activators p300 and/or CBP (CREB‐binding protein) as well as other components to transcribe a cloud of downstream target genes.^31^ Examples of which including c‐Myc and cyclin D1.^32^, ^33^ A simple outline of the components and the action model of Wnt pathway are presented in Figure 1.

Multiply dysfunctions and mutation of the pathway have suggested a strong connection with different kinds of human diseases including cancer.^34^, ^35^ It is demonstrated that the component of Wnt pathway itself may act as oncogenes or tumour suppressor genes. For example, familial adenomatous polyposis (FAP) is related to the mutation of APC,^36^ which facilitates aberrant activation of the Wnt pathway and results in adenomatous lesions owing to enhanced cell proliferation. The dysregulation of β‐catenin and APC has also been found in sporadic colon cancer and some other kinds of tumours.^37^ In addition, Wnt pathway could function as a significant anticancer target in cancer treatments.^38^, ^39^

## 4 THE INTERACTIONS BETWEEN LNCRNAS AND Wnt/β‐CATENIN SIGNALLING PATHWAY

**Figure 2:**
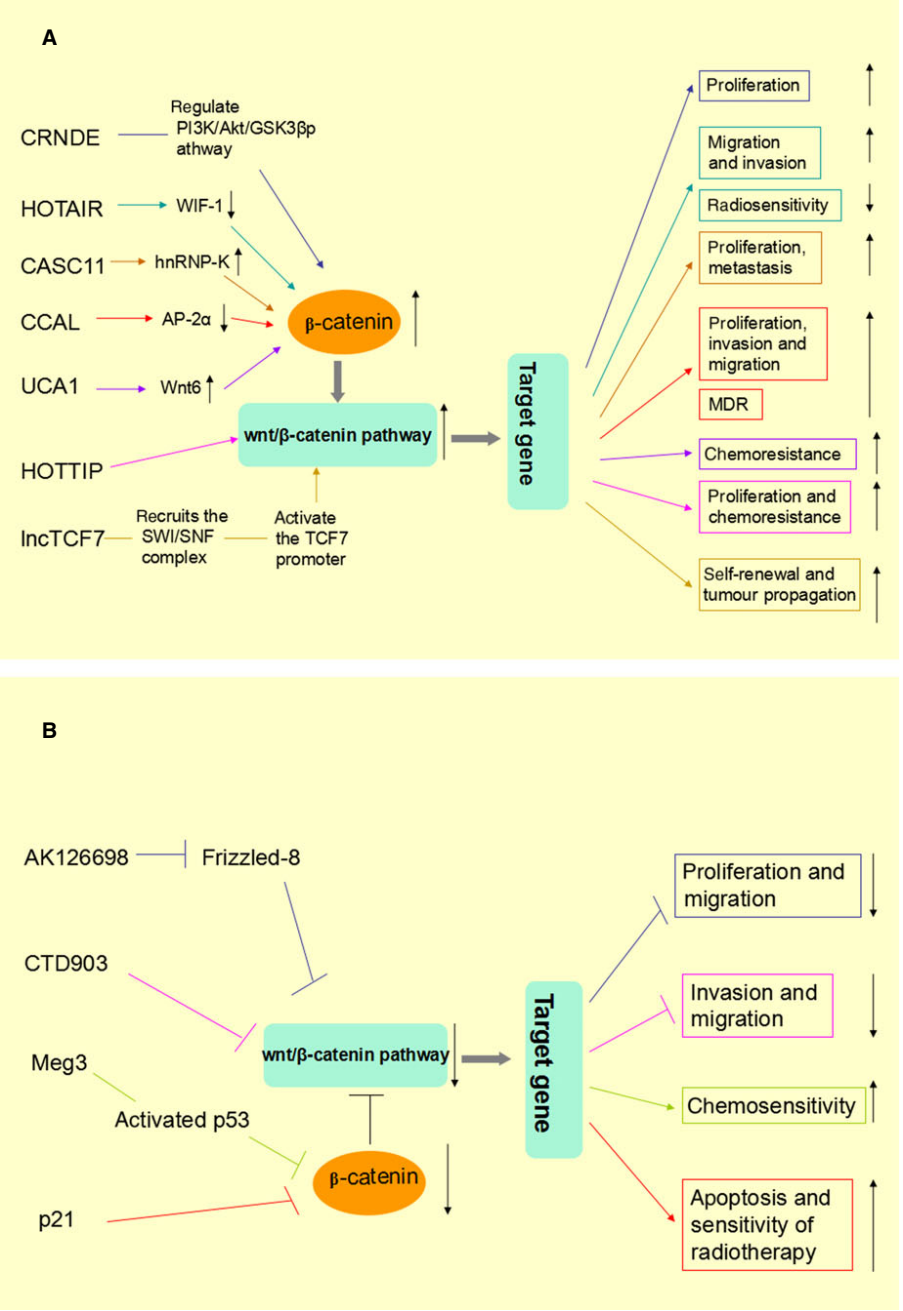
The schematic drawing showed possible regulatory network of Wnt/β‐catenin pathway‐related lncRNAs and their interactions in cancers. (A) lncRNAs which activate the Wnt pathway. (B) lncRNAs which suppress the Wnt pathway. Legend:colourized arrows indicate stimulation, colourized “T” indicates suppression, black vertical arrows indicate up‐ or down‐regulation

The Wnt pathway has been recognized as a pivotal pathway participated in the regulation of cell proliferation, tumorigenesis and progression as well as chemoresistance in various cancers.^40^, ^41^, ^42^ Dysregulation of the multifunctional protein β‐catenin, which is a crucial signalling effector in the pathway, contributes to aberrant activation of Wnt pathway.^43^ Accumulating evidence demonstrated that lncRNAs could regulate several signal pathways via interacting with specific proteins.^44^, ^45^ More recently, numerous lncRNAs have been found to target key molecules associated with Wnt pathway and affect the accumulation of β‐catenin. Then, modulating Wnt target genes expression and cancer cell functions.^46^, ^47^ For example, lncRNA CCAT2 was found to promote breast tumour progression by influencing the activity of β‐catenin, and thus, activating the Wnt pathway.^48^ These findings suggest that they may have close relationships with each other in cancer. Here, we enumerated some Wnt pathway‐related lncRNAs to explore their reciprocities in tumorigenesis and progression, drug resistance and radiosensitivity of cancers (Table 1). Their probable regulatory network was depicted in a schematic diagram (Figure 2). We also provided a perspective that a better understanding of the interactions between Wnt pathways and these lncRNAs may devise efficient strategies against tumour treatments.

**Table 1:** Overview of wnt/β‐catenin pathway‐related lncRNAs in diverse human cancers and their relations

LncRNA	Cancer Type	Associations with cancer	Interactions with Wnt pathway	References
CRNDE	Renal cell carcinoma	Promote cell proliferation	Activate	Shao et al^88^
HOTAIR	Esophageal squamous cell carcinoma	Poorer prognosis; promote migration and invasion of ESCC cells in vitro.	Activate	Ge et al^89^
	Pancreatic ductal adenocarcinoma	Reduce the radiosensitivity of PDAC cells	Activate	Jiang et al^75^
CASC11	Colorectal cancer	Promote cell proliferation and metastasis; diagnostic marker	Activate	Zhang et al^78^
CCAL	Colorectal cancer	Oncogene promote proliferation, progression, invasion and migration; MDR	Activate	Ma et al^46^
CCAT2	Breast cancer	Oncogene	Activate	Cai et al^48^
UCA1	Oral squamous cell carcinoma	Oncogene	Activate	Yang et al^90^
	Bladder cancer	Increase chemoresistance	Activate	Fan et al^47^
HOTTIP	Osteosarcoma	Increase chemoresistance	Activate	Li et al^71^
LncTCF7	Liver cancer stem cell	Oncogene	Activate	Wang et al^55^
HNF1A‐AS1	Osteosarcoma	Oncogene	Activate	Zhao et al^91^
CTD903	Colorectal cancer	Tumour suppressor inhibit cell invasion and migration	Suppress	Yuan et al^92^
AK126698	Non‐small cell lung cancer cell	Inhibit the proliferation and migration; increase apoptosis	Suppress	Fu et al^56^
Meg3	A549/DDP lung cancer cell	Enhance chemosensitivity	Suppress	XIA et al^70^
p21	Colorectal cancer	Enhance the sensitivity of radiotherapy	Suppress	Wang et al^76^

### 4.1 LncRNAs and Wnt/β‐catenin pathway in tumorigenesis and tumour progression

As is known to all, the ability to propagate continuously is one of the primary characters of cancer, and the process contains various changes in gene expression. Previously, studies into mechanisms of tumorigenesis mostly focused on protein‐coding genes. Recently, lncRNAs, with no protein‐coding ability, have been found to play widespread functions in regulating gene expression and other biological processes.^49^, ^50^ Moreover, a great many of studies have suggested that lncRNAs are essential for the regulation of tumour biology, particularly leading to abnormal expression of target gene products that contribute to the initiation and progression of numerous human cancer.^51^, ^52^, ^53^ For example, Wu et al^54^ reported that HOX transcript antisense RNA (HOTAIR), which was found to be up‐regulated in human renal carcinoma (RCC) cells, could act as an oncogenic lncRNA in promoting the proliferation and invasion of cells. Besides, the Wnt pathway is also regarded as a very significant pathway involved in tumorigenesis and progression. Studies revealed that dysregulation of the pathway was involved in different kinds of human tumours.

As the functions of lncRNAs influence many signalling pathways involved in multiple cellular processes, they could also interact with Wnt pathway through several mechanisms in tumorigenesis and progression. Recently, Shao et al reported that lncRNA CRNDE is obviously over expressed in RCC cells and the heightened CRNDE could accelerate proliferation of RCC cells via activating Wnt pathway. They found that CRNDE could somehow modulate PI3K/Akt/GSK3β pathway, then the level of β‐catenin in nucleus is elevated, which ultimately controls target gene expressions. However, the detailed mechanism remains to be expounded in further studies.^49^ In another example, Wang et al identified a long non‐coding RNA, lncTCF7, which contributes to liver cancer stem cell self‐renewal and tumour proliferation by activating Wnt signalling. Mechanism research has shown that lncTCF7 recruits the SWI/SNF complex to the promoter of TCF7 to modulate its expression, leading to activation of Wnt pathway.^55^ These evidences have indicated that the abnormal expression of Wnt pathway‐related lncRNAs plays an important role in tumour biology and could act as oncogenes. There are also several lncRNAs function as tumour suppressors. Fu et al showed that a negative regulator lncRNA AK126698, which is remarkably decreased in non‐small cell lung cancer (NSCLC) cells, can promote apoptosis and reduce the proliferation and migration of NSCLC cells by targeting Frizzled‐8 to suppress the Wnt pathway. They found that excessive expression of AK126698 down‐regulated Frizzled‐8, which in turn greatly inhibited β‐catenin expression. And this study suggests that targeting the AK126698/FZD8 interaction or disturbing AK126698 expression could become novel strategies in the treatment of NSCLC.^56^

In summary, lncRNAs activate or suppress the Wnt pathway by targeting diverse components associated with the Wnt signalling cascade to modulate downstream gene expression, revealing a strong interaction between them in tumour formation and progression.

### 4.2 LncRNAs and Wnt/β‐catenin pathway in therapeutical resistance

Chemotherapy is one of the major treatment methods of tumours. Nevertheless, chemoresistance remains an obvious obstacle against satisfactory treatment outcomes.^57^, ^58^ The mechanisms of tumour drug resistance mainly including DNA damage repair, drug efflux, mutations of drug targets and cancer cell apoptosis.^59^, ^60^ Though have been widely investigated, yet they have not been fully understood. Therefore, identification of the underlining mechanisms is of very important to develop effective strategies against drug resistance. It has recently been presumed that drug resistance is associated with genetic and epigenetic factors.^61^ Several studies have confirmed a role for lncRNAs in regulating chemoresistance.^62^, ^63^ A previous study demonstrated that lncRNA ARA may result in adriamycin resistance in cancer, by modulating numerous signalling pathways.^64^ Li et al^65^ suggested that lncRNA HOTTIP could contribute to gemcitabine resistance through regulating HOXA13 in pancreatic cancer. A novel long non‐coding RNA lncARSR was found by Qu et al^66^ who reported that lncARSR could promote sunitinib resistance in renal cell carcinoma cells and may function as a predictor and a potential therapeutic target for sunitinib resistance. These evidences emphasized the rising significance of lncRNAs in tumour chemotherapy. Besides, a variety of signalling pathways have significant contributions to the development of drug resistance. For instance, the Wnt pathway can enhance expressions of resistance factors such as MDR‐1 and surviving.^67^ Dysregulation of Wnt pathway is associated with increased chemoresistance in human cancer.^68^ For example, Su et al^69^ showed that the inhibition of secreted frizzled‐related protein is associated with chemoresistance of ovarian cancer by regulating Wnt pathway.

Considering the functions of lncRNAs and Wnt pathway in drug resistance, we then explored the underlying mechanisms and crosstalk between them in chemoresistance of cancer. As is shown, XIA et al^70^ demonstrated that MEG3 can induce cell cycle arrest and increase apoptosis by the activation of p53 as well as the inhibition of β‐catenin and survivin, which is a target gene of Wnt pathway, resulting in the suppression of Wnt pathway and enhancement of chemosensitivity in non‐small cell lung cancer. Li et al^71^ also indicated that up‐regulated HOTTIP could initiate tumorigenesis and induce drug resistance via activating the Wnt pathway, which suggested that HOTTIP may be a potential therapeutic target in osteosarcoma.

Radiotherapy is also considered as a vital component of treatment in malignant tumours. But some of them are seriously resistant to it, and the mechanism remains largely unknown, which further contributes to the poor prognosis of patients. Nowadays, several studies have indicated that some lncRNAs can affect the sensitivity of radiotherapy for human cancer.^72^, ^73^ Moreover, Wnt pathway could also enhance resistance to radiotherapy, and Wnt inhibitors may restore radiotherapy resistance.^74^ These suggest that lncRNAs may have interactions with Wnt pathway in radiotherapy of cancer. As is shown, Jiang et al reported that the expression of HOTAIR was increased with time in pancreatic ductal adenocarcinoma (PDAC) cells after X‐ray treatment. It could influence the radiosensitivity of PDAC cells via regulating the expression level of Wnt inhibitory factor‐1 (WIF‐1), which can further inhibit the activation of Wnt pathway. And the decline of HOTAIR could enhance the expression of WIF‐1, which leads to the suppression of Wnt pathway. As a consequence, decreased the proliferation, promoted the apoptosis and enhanced the radiosensitivity of PDAC cells after radiation.^75^ In another report, Wang et al found that long intergenic non‐coding RNA‐p21 (lincRNA‐p21) increases the sensitivity of radiotherapy for CRC through targeting Wnt pathway. In mechanism, the X‐ray treatment up‐regulates the expression of lincRNA‐p21 which could directly inhibit the stability and/or translation of β‐catenin. As a result, the activity of Wnt pathway is suppressed. Moreover, pro‐apoptotic protein Noxa is elevated by lincRNA‐p21. In the end, promoted cell apoptosis and enhanced the radiosensitivity for CRC.^76^

Collectively, these studies have revealed a potent interrelationship between lncRNAs and Wnt pathway in drug resistance and in radiotherapy of various cancers. Based on these findings, it is expected that further researches are needed to clarify molecular mechanisms between lncRNAs and Wnt pathway, which may act as candidates to develop novel strategies to reverse the chemoresistance and increase radiosensitivity of cancer.

### 4.3 The clinical applications of lncRNAs and Wnt/β‐catenin pathway

Detection of the specific biomarkers of tumour is one of effective methods for the early diagnosis of tumours. LncRNAs apparently exhibit a tissue‐specific expression and can be measured non‐invasively, raising the possibility that they may serve as promising biomarkers and therapeutic targets in cancer.^77^ Several studies have reported that recognition of particular lncRNAs as biomarkers may be useful in improving the diagnostic and prognosis prediction of tumours.^24^, ^25^ For instance, the lncRNA HOTAIR has been demonstrated to be connected with the increased invasion and metastasis of breast cancer cells and could serve as a predictor of overall survival and progression‐free survival. It thus may have potential clinical applications as a biomarker for breast cancer.^78^ Zhang et al reported that lncRNA CASC11 overexpression in CRC was related to TNM‐staging. It can activate Wnt pathway through targeting heterogeneous ribonucleoprotein K (hnRNP‐K) to promote proliferation and metastasis of CRC cells. Besides, CASC11 may have the ability to become a potential diagnostic biomarker and a promising therapeutic target for CRC.^79^ Another typical clinical application example is lncRNA PCA3, which is highly expressed in prostate cancer, can be detected in urine samples of patients and has been demonstrated to have greater sensitivity and specificity for the early diagnosis of prostate cancer than the normally used prostate‐specific antigen (PSA) blood test.^80^

Considering the functions of lncRNAs in cancer, recently, emerging studies have indicated that biological therapies targeting these lncRNAs by the use of small interfering RNAs, ribozymes, antisense oligonucleotides, and by the small molecule inhibitors may be novel approaches to fight against cancer.^81^, ^82^ For example, CCAL is an oncogenic lncRNA that promotes the tumorigenesis and progression of colorectal cancer (CRC) through activation of Wnt pathway. Overexpression of CCAL was related to poor survival and poor response to adjuvant chemotherapy. CCAL knockout inhibited CRC cell proliferation, migration and invasion and induced cell apoptosis in vitro and in vivo, which make it a potential therapeutic target for CRC.^46^ The lncRNA UCA1 is highly up‐regulated and may be a reliable biomarker in bladder cancer. RNA interference‐mediated down‐regulation of UCA1 inhibited proliferation, migration and invasion of the bladder cancer cell, suggesting it can be a novel therapeutic target.^83^ Nevertheless, the clinical use of the therapy targeting these lncRNAs is still in its infancy, there are many obstructions need to be solved before the clinical popularization of the lncRNA‐based treatments.

On the other hand, several crucial components of the Wnt pathway could act as potential biomarkers for cancer diagnosis or prognosis and have been recognized as innovative targets for cancer therapy.^38^, ^39^ Arend RC et al found that niclosamide could increased cell apoptosis and reduce cellular proliferation via inhibiting the Wnt pathway. Targeting the Wnt pathway may become a novel strategy for the treatment of ovarian cancer.^84^ Currently, several antibodies and small molecular inhibitors have been investigated and are undergoing pre‐clinical or clinical trials, such as OMP‐18R5 (Anti Fzd7 antibody), OMP54F28 (Soluble Fzd decoy receptor), PRI‐724 (Inhibitor of TCF‐CBP interaction), which have been described by Zhan T in the 2016.^85^ However, to date, there are no suitable candidates available in clinic that specifically and efficiently targets this pathway, mainly because of noteworthy side effects. And more efforts are needed to precisely regulate the pathway before their clinical applications.

## 5 CONCLUSIONS AND PERSPECTIVE

LncRNAs are a class of non‐protein–coding RNAs, which play important roles in diverse biological processes.^86^, ^87^ Accumulating evidences have strongly confirmed that lncRNAs have close interactions with Wnt pathway in tumorigenesis, development and therapy resistance. As is shown, many Wnt pathway‐related lncRNAs are aberrantly expressed in various cancers. They are useful as diagnostic indicators or prognosis prediction of tumours and can act as oncogenes or tumour suppressor genes. In mechanism, these lncRNAs can activate or suppress the Wnt pathway by targeting different molecules associated with the pathway. Such as Wnt ligands, Wnt inhibitors, receptors, β‐catenin, components of the degradation complex and transcription factors as well as elements in other signalling pathways, subsequently, regulating the expression of Wnt target genes involved in cell multiplication, migration, tumorigenesis and therapy resistance.^43^ Meanwhile, the expression of lncRNAs can also be influenced by Wnt pathway. For example, a recent report demonstrated that c‐Myc, a downstream gene of Wnt pathway, could combine with the promoter region of CASC11 and improve histone acetylation to increase CASC11 expression in CRC.^79^

To summarize, the close interaction between lncRNAs and Wnt/β‐catenin signalling pathway seems to play a significant role in tumorigenesis, progression and influencing the response of cancer cells to treatments. Potential therapies targeting lncRNAs or Wnt pathway have been mentioned in some documents. However, further studies about the underlying mechanisms are needed to promote an efficient therapeutic strategy into a reality by targeting lncRNAs and/or Wnt pathway in cancer.

## CONFLICT OF INTEREST

The authors declare that we have no conflict of interest.
